# Treatment outcome is associated with pre-treatment connectome measures across psychiatric disorders − evidence for connectomic reserve?

**DOI:** 10.1016/j.nicl.2025.103870

**Published:** 2025-08-18

**Authors:** Chris Vriend, Sophie M.D.D. Fitzsimmons, Inga Aarts, Aniek Broekhuizen, Ysbrand D. van der Werf, Linda Douw, Henny A.D. Visser, Kathleen Thomaes, Odile A. van den Heuvel

**Affiliations:** a Amsterdam UMC, Vrije Universiteit Amsterdam, Department of Psychiatry, de Boelelaan 1117, Amsterdam, the Netherlands; b Amsterdam UMC, Vrije Universiteit Amsterdam, Department of Anatomy and Neurosciences, de Boelelaan 1117, Amsterdam, the Netherlands; c Compulsivity, Impulsivity and Attention, Amsterdam Neuroscience, de Boelelaan 1117, Amsterdam, the Netherlands; d Sinai Centrum, Arkin, Amstelveen, the Netherlands; e Amsterdam UMC, Universiteit van Amsterdam, Department of Psychiatry, Meibergdreef 9, Amsterdam, the Netherlands

**Keywords:** Neuroimaging, Psychotherapy, Transdiagnostic, Connectome, Obsessive-compulsive disorder, Posttraumatic stress disorder

## Abstract

Predicting treatment efficacy in psychiatric disorders remains challenging, despite the availability of effective interventions. Previous studies suggest a link between pre-treatment brain network characteristics and treatment efficacy in individual disorders, but cross-disorder investigations are lacking.

We analyzed pre-treatment MRI data from 177 individuals (113 females) with either obsessive–compulsive disorder (OCD) or post-traumatic stress disorder with comorbid personality disorders (PTSD) that received different non-pharmacological treatments. Using diffusion and resting-state MRI, we constructed structural, functional, and multilayer connectomes and calculated network measures for network integration (e.g. global efficiency, eccentricity), segregation (modularity) and their balance (small-worldness). We assessed the relationship between these pre-treatment network measures, and treatment improvement using mixed-model and Bayesian analyses. We also compared psychiatric cases with healthy controls and investigated associations between clinical response and treatment-induced changes in network measures.

Across disorders and treatments, psychiatric cases showed a 41.6 ± 29.6 % symptom improvement (62 % response rate) after treatment. They also showed pre-treatment differences in functional and multilayer network topology compared to healthy controls. Symptom improvement was associated with pre-treatment functional (P = 0.04) and structural small-worldness (P = 0.01), and multilayer eccentricity (P = 0.01), while responders had higher functional modularity (P = 0.02). Results were robust across trials and treatments, when adjusting for medication status and showed high credibility in Bayesian analyses. Network change associations with treatment response were only modest.

These results show that pre-treatment connectome characteristics are related to treatment response, regardless of treatment and psychiatric disorder, and suggest that individual differences in intrinsic features of the human connectome underlie amenability to treatment.

## Highlights

• Can brain network measures help explain who benefits from treatment?
• Symptom improvement was related to baseline structural & functional network measures.
• This was regardless of psychiatric disorder, received treatment & medication status.
• Individual network variation across disorders relates to treatment outcome.
• Brain network markers may predict treatment success better than diagnostic labels.

## 1 Introduction

Deficits in cognitive control – the ability to regulate one's thoughts, emotions and behavior – underlie many of the cognitive and psychological symptoms in individuals with a psychiatric disorder (McTeague et al., 2017, Millan et al., 2012). For example, anxiety provoking thoughts (obsessions) and ritualistic behaviors (compulsions) in patients with obsessive–compulsive disorder (OCD), or traumatic flashbacks in post-traumatic stress disorder (PTSD) are associated with cognitive control deficits (Aupperle et al., 2012, Fitzgerald et al., 2021, Grisham and Williams, 2009). Across psychiatric disorders, these cognitive control deficits are accompanied by disturbed communication within the brain ‘connectome’ (Crossley et al., 2014, Griffa et al., 2013, Lord et al., 2017, Sha et al., 2019): the arrangement of structural and functional connections between brain areas that form a complex network (Bullmore and Sporns, 2009). According to graph theory, the normal architecture of the connectome is governed by two fundamental principles: segregation (processing in specialized subsystems, e.g. visual system) and integration (i.e. exchange between subsystems) (Bullmore and Sporns, 2009). A key component of this architecture is also a modular network structure with sparse cross-modular long range connections (Lord et al., 2017). It has previously been suggested that the development and severity of brain-related disorders is associated with an imbalance between segregation and integration (van den Heuvel and Sporns, 2019). This view is supported by studies that have suggested common connectome dysfunction across brain-related disorders, both in psychiatry, (Lord et al., 2017, Sha et al., 2019, Spronk et al., 2021) and neurology (de Lange et al., 2019, Hansen et al., 2022). Nevertheless, few head-to-head comparisons between brain-related disorders have been conducted. Some studies have investigated cross-disorder differences in local brain anatomy (Hansen et al., 2022). Others showed structural dysconnectivity of brain areas involved in cognitive control that are also vital for network integration,(de Lange et al., 2019) or common functional patterns across psychiatric disorders with only subtle differences with healthy controls (Spronk et al., 2021).

Treatments, such as psychotherapy and neurostimulation, can enhance cognitive control and have been shown to normalize connectome dysfunction (Davis et al., 2023, Nord et al., 2021, Szeszko and Yehuda, 2019). Unfortunately, however, not everyone (fully) benefits from these treatments (Kim, 2018), with significant risk of chronicity. Predicting who will respond to treatment remains, however, challenging. Some studies have been able to predict the outcome of cognitive behavioral therapy (Cyr et al., 2020, Gottlich et al., 2015, Zhutovsky et al., 2019), and neurostimulation (Avissar et al., 2017, Ge et al., 2020), but none of these predictors are currently used in the clinic, mainly due to replication failures (Cohen et al., 2021, Colvonen et al., 2017). Indeed, studies on imaging markers for treatment response in OCD (Cao et al., 2021, Cyr et al., 2020, Pagliaccio et al., 2020, Reggente et al., 2018, Shi et al., 2021) or PTSD (Fonzo et al., 2021, Korgaonkar et al., 2020, van Lutterveld et al., 2022, Zhutovsky et al., 2019, Zhutovsky et al., 2021) have been mixed in part due to the heterogeneity of the methods; see also (Cao-Noya et al., 2025, Moreau et al., 2025) for reviews), and only few used functional (van Lutterveld et al., 2022) or structural (Cao et al., 2021) connectome measures.

Motivated by the previous findings on shared deficits across psychiatric disorders in both cognitive control and the organizational structure of the connectome, this study will investigate whether cross-disorder connectome characteristics are associated with treatment outcome of non-pharmacological, cognitive control enhancing treatments. Previous studies have already identified modularity as a potential predictor for treatment efficacy in single treatments and disorders (Gallen and D’Esposito, 2019), but to our knowledge this has not yet been investigated across disorders and treatments in a single study. Identification of network markers that predict treatment outcome across psychiatric disorders not only fits with the dimensional approach of Research Domain Criteria (RdoC) (Insel and Cuthbert, 2015), and allows leveraging of larger, better powered and diverser datasets, it can also enhance our understanding of commonalities in treatment resistance and help tailor mechanism-based treatments by focusing more on individual variation in network topology along a continuum rather than on symptom-based diagnostic labels. We associated pre-treatment network measures with treatment response by combining data from four clinical trials with pre- and post-treatment structural and functional MRI in two psychiatric disorders (OCD and PTSD). These clinical trials investigated nine different treatments (see methods section) involving different forms of psychotherapy (some in combination with neurostimulation). We hypothesized that efficacy of treatment was associated with pre-treatment multiscale network measures of segregation and integration, regardless of the psychiatric disorder or treatment (see also our pre-registration: osf.io/p7bcn). We additionally investigated cross-disorder associations between treatment-induced network changes and treatment response. In these analyses we hypothesized that successful treatment would be associated with changes in network integration and segregation. Given the novelty of this research topic, we did not specify hypotheses about the directionality or the importance of either modality.

## 2 Experimental procedures

### 2.1 Participants and interventions

This project included participants from several completed clinical trials: ARRIBA (NCT03929081), TIPICCO (NCT03667807), and PROSPER (NCT03833531/NCT03833453). Details are provided in the supplements and in the clinical trial papers (Fitzsimmons et al., 2024, Snoek et al., 2025, van den End et al., 2024, Wolf et al., 2024). Briefly, ARRIBA involved OCD participants randomized to receive either cognitive behavioral therapy (CBT) with exposure and response prevention (ERP) or inference-based CBT (I-CBT). TIPICCO participants with OCD received ERP combined with repetitive transcranial magnetic stimulation (rTMS). Both trials measured OCD symptom severity with the Yale-Brown Obsessive-Compulsive Scale (YBOCS) before and after treatment. The PROSPER study comprised two trials for individuals with PTSD and a comorbid personality disorder: PROSPER-B and PROSPER-C. PROSPER-B participants received eye movement desensitization and reprocessing (EMDR) alone or with dialectical behavioral therapy (DBT). PROSPER-C participants received imagery rescripting alone or with schema therapy. Both trials used the clinician-administered PTSD interview for DSM-5 (CAPS-5) to assess PTSD severity pre- and post-treatment. All participants received active treatment and provided informed consent. All trials were approved by the VU Medical Center Medical Ethical Committee. Inclusion/exclusion criteria are in the supplements. All TIPICCO, but only a subset of ARRIBA, and PROSPER participants underwent MRI. Those with baseline diffusion MRI, resting-state functional MRI, and pre/post-treatment clinical measures were included in the analysis. Non-compliant individuals that underwent a post-treatment clinical assessment were included in all analyses (i.e. intention-to-treat sample). A matched healthy control group, free of psychopathology, medication and neurological disorders, that were scanned on the same MRI scanner (see below) was selected from the ARRIBA, TIPICCO, PROSPER, and additional studies, COGTIPS (van Balkom et al., 2022), and the global OCD study (Pouwels et al., 2023).

### 2.2 Image acquisition and preprocessing

All participants were scanned on a GE Discovery MR750 3 T scanner (General Electric, Milwaukee, U.S) using a 32-channel head coil with harmonized sequences, including a 3D T1-weighted MRI, 10 min eyes closed resting-state fMRI (rsfMRI) in a dark room and multi-shell diffusion MRI (dMRI). Acquisition parameters and preprocessing steps are provided in the supplements. FMRI scans with a mean FD > 0.5 mm were excluded. DMRI scans were visually inspected for residual (motion) artifacts and if necessary excluded.

### 2.3 Connectome construction and network measures

We used the Schaefer 300P7N atlas and 14 individually segmented subcortical areas (FreeSurfer 7.3.2) to extract time series from the preprocessed, denoised functional images. Following recommendations for consistent network reconstruction (Luppi et al., 2024, Ran et al., 2020) we applied Pearson correlations, absolutized negative correlations, and used Orthogonal Minimal Spanning Tree (OMST) thresholding to create a 314x314 weighted functional connectome. The same parcellation and OMST thresholding algorithm were used to construct a weighted structural connectome from the dMRI tractogram. To build a multilayer connectome, we calculated the minimum spanning tree (MST) of both structural and functional connectomes and created interlayer links (with weight 1) that connect the same nodes across structural and functional layers. The use of MST prevents differences in link density and connection strength between layers and participants (Tewarie et al., 2015).

We calculated several widely used global network measures from the structural, functional and multilayer connectomes that capture the integrative and segregative capacity of connectomes. Global efficiency, modularity, average participation coefficient (PC) and small-worldness were calculated for the structural and functional connectomes. Global efficiency measures how efficiently information can be transferred through a network. Modularity represents how divided the network is into interconnected groups of nodes (i.e., modules) with a higher modularity indicating a more modular structure. PC provides a measure for the connectivity of a node with its own module relative to other modules. The average PC therefore represents the interconnectedness of the network across modules. Small-worldness represents the ratio between local clustering and global integration. For the multilayer, we calculated the average eccentricity and eigenvector centrality. Eccentricity represents the maximum distance between one node and all others, with the average eccentricity representing how far a signal has to travel to cross the network. Eigenvector centrality measures how influential (i.e. central) a node is for network communication. Higher average eigenvector centrality represents a more integrated network structure with many central nodes. Further definitions and details are provided in the supplements.

On the mesoscale, we investigated structural and functional connectivity strength between eight ‘systems’, seven Yeo cortical networks (Yeo et al., 2011) – Default mode (DMN), Frontoparietal (FPN), Dorsal attention (DAN), Ventral Attention (VAN), Somatomotor (SMN), Limbic and Visual Network – and 14 subcortical regions. We additionally calculated the average PC of the nodes in each of the systems for the structural and functional connectomes and the average eccentricity and eigenvector centrality for the multilayer connectome (as PC is not suitable for multilayer connectomes). More details are provided in the supplements.

### 2.4 Statistical analysis

Analyses were pre-registered with the Open Science Framework (osf.io/p7bcn). Because the trials used different scales as outcome measures, we defined treatment outcome across disorders as the percentage improvement from pre-to-post-treatment. We additionally defined treatment responders and non-responders based on the established cut-off score for OCD (Y-BOCS: ≥35 % improvement (Mataix-Cols et al., 2016), and ≥1 pooled SD improvement on the CAPS-5 as no uniform response definition for PTSD treatment exists (van den End et al., 2024).

We performed univariate mixed model analyses with pre-treatment global network measures (single or multilayer) as the dependent variable and percentage change or responder/non-responder as the independent variable, with and without covariates for age and sex. To capture consistent and universal markers of treatment response we only adjusted for clinical trial or treatment in separate sensitivity analyses using random intercepts. These null hypothesis significance testing (NHST) models were evaluated at p < 0.05, and as pre-registered, the global measures were not corrected for multiple comparisons.

At the mesoscale, we used Bayesian multilevel modeling to include information across all systems into one statistical model. Treatment outcome was associated with structural and functional connectivity between the eight systems using Matrix-based Bayesian analyses (Chen et al., 2019a) and with averaged PC measures (single layers) and averaged eccentricity and eigenvector centrality measures (multilayer) of the eight systems using Region-Based Analysis Program through Bayesian Multilevel Modeling (RBA) (Chen et al., 2019b). The advantages of these Bayesian multilevel approaches for neuroimaging data, including more transparent reporting and simultaneous integration of related measures from the same brain, are detailed in the supplements. Bayesian models produce posterior distributions, with the area under the curve to the right or left of the zero-effect line representing the effect's credibility, expressed as the positive posterior probability (P+). The distance of the posterior mode from the zero-effect line indicates the effect's magnitude. While inferences are based on the full posterior distribution, consistent with previous studies (Broekhuizen et al., 2023, Chen et al., 2019b, Dzinalija et al., 2024, Madzime et al., 2025), we only report effects with P+ <0.10 or >0.90 in the main text and classified effect credibility as *moderate* (P+ between [1-]0.05 and [1-]0.10), *strong* (P+ between [1-]0.01 and [1-]0.05), and *very strong* (P+ <0.01 or >0.99). We also performed exploratory network-based statistics (NBS) on the full 314x314 structural or functional connectomes to identify clusters of edges that formed an interconnected subnetwork that are associated with treatment outcome at a T-threshold of 3.1 (default).

Additionally, using similar statistical models, we compared the psychiatric samples at baseline with the pooled healthy control sample to investigate the pre-treatment starting point relative to healthy controls. We used the MatchIt R package to select age and sex matched controls from a pool of N = 158.

To determine how single-layer and multilayer topology of the network change with successful treatment we used models that deviated from the pre-registration plan. This is because we realized during the analyses that the pre-registered univariate mixed model analyses with post-treatment network measure as dependent variable, treatment outcome as independent variable and pre-treatment network measure as covariate did not allow us to capture the changes in clinical symptoms or response status and changes in network measures as intended. For the percentage change models we therefore z-transformed the individual-centered pre-treatment and post-treatment clinical severity scores (to adjust for differences in scales between trials) and subsequently associated these values with the pre-to-post treatment global network measures in a hierarchical linear regression model with ‘participant’ as a fixed effect. For the responder vs non-responder analyses we used mixed model analyses with a random intercept for participant, the network measure as dependent variable and an interaction term for time and response status (along with their main effects), while adjusting for the pre-treatment value of the network measure, age and sex. These models and the reasons for the deviations are further explained in the supplements. Lastly, leave-one-trial out and leave-one-treatment out (collectively called leave-one-sample-out [LOSO]) validation were performed to determine the robustness of the findings. For the global topology measures we calculated the harmonic mean p-value across folds which can handle dependencies between p-values and is less stringent than a Bonferroni correction (Wilson, 2019). For the Bayesian analyses we report the median and range in P+ values across folds.

### 2.5 Code availability

Preprocessing and analyses code is available at: github.com/chrisvriend/core-multi.

## 3 Results

### 3.1 Demographics, clinical information

Of the 200 individuals that completed the interventions, 23 had to be excluded because they did not consent to the re-use of their data, leaving data from 177 for the current project. Forty-three participants did not return for the post-treatment MRI (in part because of COVID regulations), leaving 134 for the pre-to-post treatment analyses (Supplementary Table 1). Some additional functional or diffusion MRI scans had to be excluded because of missing or low-quality data (see flowchart in Supplementary Fig. 1). The prediction sample included 172 individuals with diffusion MRI, 167 with functional MRI and 162 individuals with both. The demographic and clinical details of the prediction sample are presented in Table 1, showing on average a 41.6 (±29.6) percent treatment-induced symptom improvement across trials and a 61.6 % response rate. Before treatment, 87 were on psychotropic medication. Medicated and non-medicated individuals did not differ in percentage improvement (t_(155)_ = 0.26, P = 0.80) or response rate (χ_(1)_ = 0.024, P = 0.99). There were no differences in frame-wise displacement during fMRI between the treatment groups (F_(8,166)_ = 0.69, P = 0.70). MatchIt selected 143 healthy controls that were matched on age (Welch’s t_(283.84)_ = 0.77, P = 0.44) and sex (χ_(1)_ = 3.3, P = 0.07) and they showed no differences in frame-wise displacement (F_(1,312)_ = 0.08, P = 0.78).

**Table 1:** Demographic and clinical characteristics.

Sample	Treatment	N	Age (yrs)	T0 sev.#	T1 sev.#	% improvement	Sex (F/M)	Responder (no/yes)	Med (no/yes)	FD fMRI
Total		177	36.7 ± 11.7			41.6 ± 29.6	113/64	68/109	88/87*	
ARRIBA	CBT-ERP	28	30.4 ± 9.1	24 ± 3.7	12.2 ± 7.2	48.1 ± 33.6	15/13	10/18	15/13	0.12 ± 0.05
	I-CBT	35	34 ± 10.5	24.2 ± 4.4	16.3 ± 7.1	32.9 ± 25.3	19/16	18/17	19/16	0.14 ± 0.06
TIPICCO	DLPFC	17	41.5 ± 12.2	28.5 ± 5.7	16.8 ± 8.1	41.9 ± 22.8	11/6	6/11	5/12	0.15 ± 0.09
	preSMA	20	32.5 ± 14	29 ± 3.5	19.6 ± 7.9	33.2 ± 25	11/9	12/8	7/13	0.13 ± 0.06
	vertex	18	40.1 ± 11.6	26.6 ± 4	15.6 ± 4.8	40.5 ± 19.5	12/6	6/12	9/9	0.12 ± 0.05

PROSPER-B	EMDR	14	36.1 ± 10.9	47.8 ± 10.2	30.8 ± 15.6	37.6 ± 28.2	13/1	4/10	8/6	0.12 ± 0.04
	EMDR + DBT	11	37.7 ± 7.2	41.7 ± 11	23.9 ± 14.4	41.9 ± 35.1	9/2	4/7	6/4	0.11 ± 0.04
PROSPER-C	IR	16	45.4 ± 11.4	37.8 ± 11.3	19 ± 18.7	51.2 ± 43.3	10/6	4/12	8/7	0.15 ± 0.12
	IR + SFT	18	40.3 ± 10.9	45.3 ± 9.9	22.1 ± 16.6	53.2 ± 29.5	13/5	4/14	11/7	0.14 ± 0.06

Healthy controls	−	143	37.8 ± 13.4	−	−	−	76/67	−	−	0.13 ± 0.06

Scores are presented as mean ± standard deviation, unless otherwise indicated. # score represents the severity on the Yale-Brown Obsessive-compulsive Scale (Y-BOCS) for the ARRIBA and TIPICCO trials, and the clinician-administered PTSD scale for DSM-5 (CAPS-5) for the PROSPER trials. Medication is operationalized as using selective serotonin reuptake inhibitors (SSRI’s), serotonin-norepinephrine reuptake inhibitors (SNRI), tricyclic antidepressants (TCAs), antipsychotics or mood stabilizers at the time of the T0 MRI scan. *data was missing for two cases. Abbreviations: CBT-ERP = cognitive behavioral therapy (CBT) with exposure and response prevention (ERP) therapy, IBA = inference based approach, DLPFC = repetitive Transcranial Magnetic Stimulation (rTMS) to the dorsolateral prefrontal cortex in combination with ERP, preSMA = rTMS to the pre-supplementary motor area in combination with ERP, vertex = rTMS to the vertex in combination with ERP, EMDR = eye-movement desensitization and reprocessing, EMDR + DBT = EMDR and dialectical behavioral therapy, IR = imagery rescripting therapy, IR + SFT = imagery rescripting and schema focused therapy.

### 3.2 Pre-treatment case-control differences

**Fig. 1:**
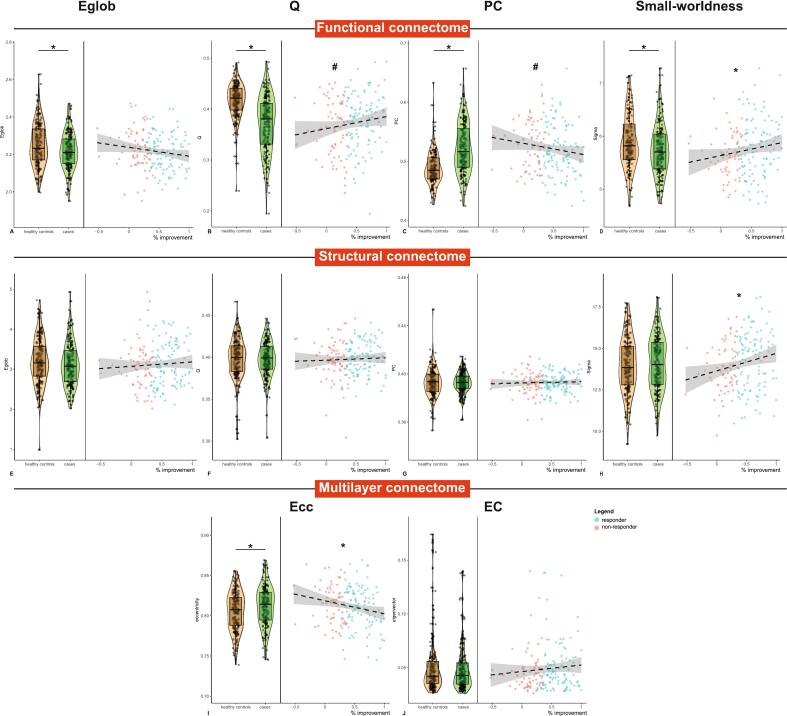
Case-control differences and associations with treatment outcome of pre-treatment global network measure. Violin plots show the distribution of the functional (a–d), structural (e–h) and multilayer (i–j) network measures. There were significant between-group differences in all functional network measures and the average multilayer eccentricity. Scatterplots show the associations between pre-treatment network measures and percentage symptom improvement, with positive values representing improvement after treatment. individual dots are colored according to whether someone was a responder (green) or non-responder (red). Percentage symptom improvement was significantly associated with functional (d) and structural (h) small-worldness and multilayer average eccentricity (i). Responders and non-responders also showed significant differences in functional modularity (b) and average participation coefficient (c). * significant association between percentage symptom improvement and the pre-treatment network measures. # significant difference in pre-treatment network measure between treatment responders and non-responders. abbreviations: Eglob = global efficiency, Q = modularity, PC = average participation coefficient, Ecc = average eccentricity, EC = average eigenvector centrality. (For interpretation of the references to color in this figure legend, the reader is referred to the web version of this article.)

Case-control analyses showed significantly lower global efficiency (B[SE] = −3.53[1.2], P = 0.004), modularity (B[SE] = −4.6[0.6], P < 0.001) and small-worldness (B[SE] = −14.7[5.7], P = 0.01) and higher average PC (B[SE] = 3.5[0.4], P < 0.001) in the patient population for the functional but not structural connectome and higher multilayer eccentricity (B[SE] = 0.7[0.3], P = 0.02; see Fig. 1 and Supplementary Tables 2–3). These results were also evident at the system level (Supplementary Figs. 2–3). These differences suggest that psychiatric cases have suboptimal integrative and segregative properties of the functional connectome and lower multilayer integrative capacity.

### 3.3 Treatment outcome and pre-treatment network measures

Percentage symptom improvement was positively associated with pre-treatment small-worldness of the functional (B[SE] = 24.4[12.6], P = 0.04) and structural connectome (B[SE] = 105.4[41.4], P = 0.01) and negatively with multilayer eccentricity (B[SE] = −1.6[0.7], P = 0.01). Compared with non-responders, responders showed a higher modularity (B[SE] = 1.8[0.9], P = 0.02) and lower average PC (B[SE] = −1.3[0.7], P = 0.03) of the functional connectome (Table 2). Results were robust against LOSO validation and sensitivity analyses adjusting for trial or treatment showed similar results (Supplementary Table 4). Other global measures were not associated with treatment response (Supplementary Table 5).

**Table 2:** Pre-treatment global connectome measures and treatment response.

	Crude model			Adjusted model*			LOSO	
% change	B [SE]	95 % CI	P	B [SE]	95 % CI	P	Trial (harm.P)	Treatment (harm. P)
Functional connectome								
Eglob (x 10^−3^)	−4.700 [2.600]	−9.8|0.4	0.068	−4.500 [2.600]	−9.6|0.5	0.077	0.033	0.001
Q_yeo_ (x10^−2^)	2.400 [1.400]	−0.4|5.2	0.097	2.500 [1.400]	−0.2|5.2	0.074	0.021	0.001
PC_yeo_ (x10^−2^)	−1.900 [1.100]	−4.1|0.3	0.084	−2.000 [1.100]	−4.1|0.1	0.059	0.013	<0.001
Small-worldness (x10^−2^)	24.400 [12.600]	−0.5|49.4	0.055	25.400 [12.400]	0.9|49.9	0.042	0.009	<0.001

Structural connectome								
Eglob (x 10^−4^)	11.000 [13.900]	−16.3|38.4	0.428	11.000 [13.900]	−16.5|38.5	0.433	0.49	0.655
Q_yeo_ (x 10^−2^)	0.300 [0.500]	−0.8|1.4	0.601	0.200 [0.500]	−0.8|1.3	0.652	0.827	0.898
PC_yeo_ (x 10^−2^)	0.100 [0.200]	−0.3|0.5	0.632	0.100 [0.200]	−0.3|0.6	0.565	0.828	0.922
Small-worldness (x 10^−2^)	105.400 [41.400]	23.7|187.1	0.012	99.000 [39.500]	21.1|176.9	0.013	<0.001	<0.001

Multilayer connectome								
EC (x 10^−2^)	0.600 [0.600]	−0.5|1.7	0.290	0.600 [0.600]	−0.5|1.7	0.282	0.401	0.253
Ecc (x 10^−2^)	−1.600 [0.700]	−2.9|−0.3	0.013	−1.600 [0.600]	−2.9|−0.4	0.013	<0.001	<0.001

Responders vs non-responders								
Functional connectome								
Eglob (x 10^−3^)	−2.500 [1.600]	−5.6|0.6	0.117	−2.500 [1.600]	−5.6|0.6	0.119	0.073	0.007
Q_yeo_ (x 10^−2^)	1.800 [0.900]	0.1|3.5	0.042	1.900 [0.900]	0.3|3.6	0.025	0.003	<0.001
PC_yeo_ (x 10^−2^)	−1.300 [0.700]	−2.6|0.00	0.054	−1.400 [0.700]	−2.7|−0.1	0.031	0.005	<0.001
Small-worldness (x 10^−2^)	12.600 [7.800]	−2.9|28	0.110	13.800 [7.700]	−1.3|29	0.074	0.031	0.001

Structural connectome								
Eglob (x 10^−4^)	−0.200 [8.500]	−17|16.5	0.977	−0.000 [8.600]	−16.9|16.9	0.997	0.71	1.00
Q_yeo_ (x 10^−2^)	0.100 [0.300]	−0.5|0.8	0.707	0.100 [0.300]	−0.5|0.8	0.712	0.813	0.98
PC_yeo_ (x 10^−2^)	0.100 [0.100]	−0.1|0.4	0.275	0.200 [0.100]	−0.1|0.4	0.251	0.342	0.175
Small-worldness (x 10^−2^)	37.600 [25.700]	−13.1|88.2	0.145	36.800 [24.500]	−11.5|85.1	0.135	0.057	0.01

Multilayer connectome								
EC (x 10^−2^)	0.600 [0.400]	−0.1|1.3	0.104	0.600 [0.400]	−0.1|1.3	0.115	0.083	0.007
Ecc (x 10^−2^)	−0.400 [0.400]	−1.2|0.5	0.378	−0.300 [0.400]	−1.1|0.5	0.432	0.427	0.668

*Corrected for age and sex. Abbreviations: LOSO = leave-one-sample-out – either one of the trials or one of the treatments. Eglob = global efficiency, Q = modularity quality index, PC = participation coefficient, EC = eigenvector centrality, Ecc = eccentricity.

**Fig. 2:**
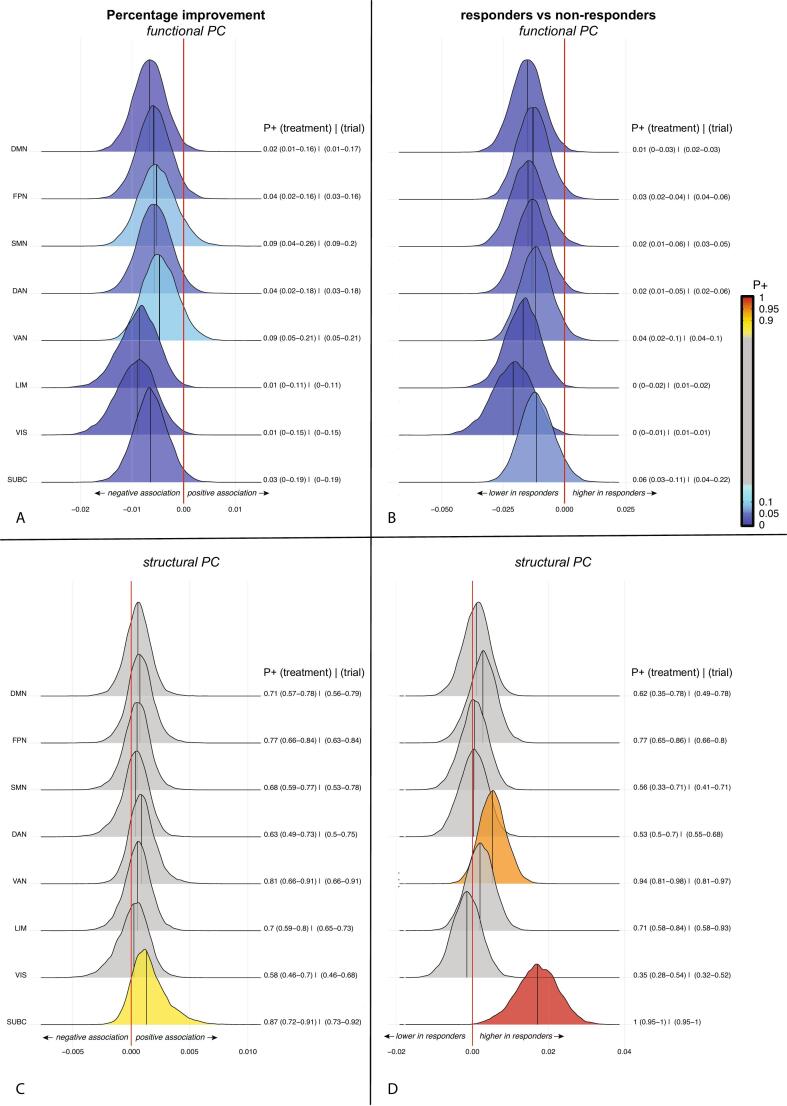
Bayesian posterior distribution plots of the association between structural and functional participation coefficient of eight brain systems and percentage change (a,b) or response status (c,d). The posterior distribution communicates the credibility of an effect. Positive posterior probabilities (*P+*) are shown next to each distribution and color coded. The values between brackets indicate the range of P+ values across leave-one-treatment-out, or leave-one-trial-out folds to show the robustness of the results. *P+* values ≥0.90 indicate moderate to very high credibility for a positive effect, P+ ≤0.10 indicate moderate to very high credibility for a negative effect. The meaning of the direction of effects are shown next to the red zero-effect line. There was credible evidence for (a) negative associations between functional participation coefficient and percentage symptom improvement, and (b) lower functional participation coefficient in responders compared with non-responders across all eight systems. With the exception of a higher structural participation coefficient of the ventral attention network and subcortical structures in responders compared with non-responders (d), there was no credible evidence for associations between treatment outcome and structural participation coefficient. abbreviations: PC = participation coefficient, DMN = default mode network, FPN = frontoparietal network, SMN = somatomotor network, DAN = dorsal attention network, VAN = ventral attention network, LIM = limbic network, VIS = visual network. SUBC = subcortical structures. (For interpretation of the references to color in this figure legend, the reader is referred to the web version of this article.)

**Fig. 3:**
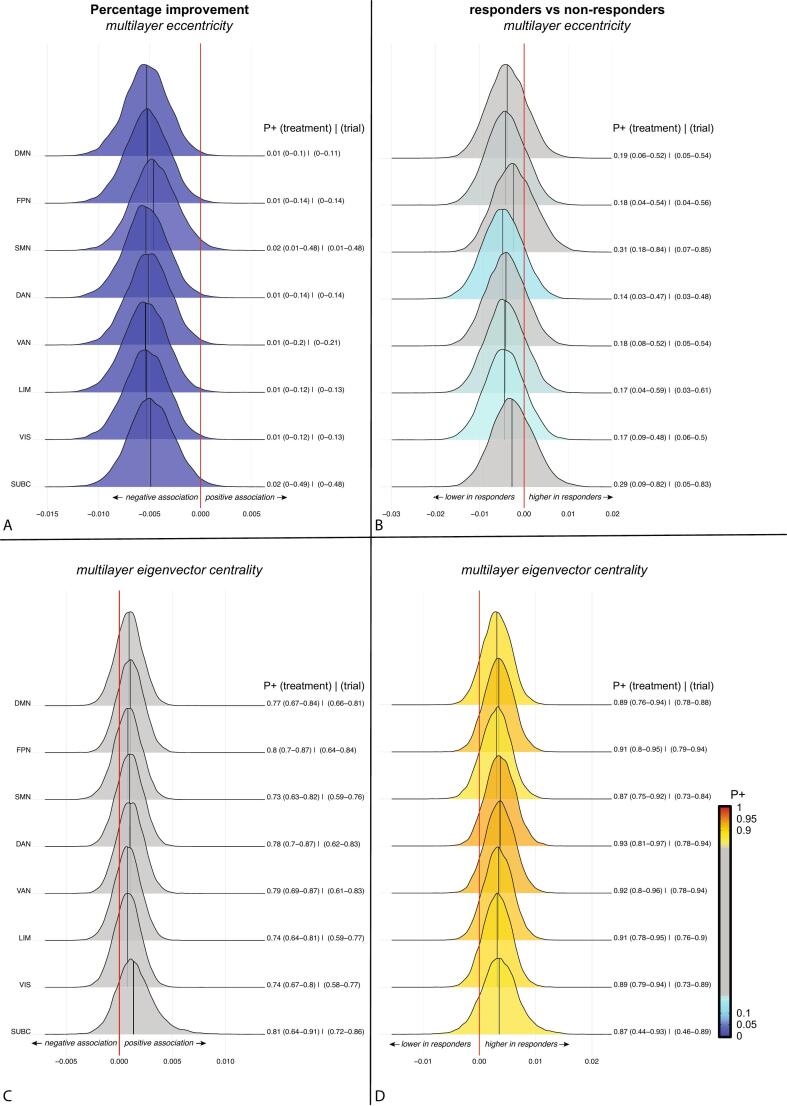
Bayesian posterior distribution plots of the associations between multilayer eigenvector centrality and eccentricity of eight brain systems and percentage change (a,b) or response status (c,d). See the legend of Fig. 2 for an explanation of the posterior distribution plots. There was credible evidence for (a) negative associations between multilayer eccentricity and percentage symptom improvement across all eight systems, but (b) no credible evidence for differences between responders and non-responders. There was also no credible evidence for an association between (c) symptom improvement and multilayer eigenvector centrality. (d) Multilayer eigenvector centrality was higher in responders compared with non-responders in all eight networks but only the dorsal and ventral attention network as well as frontoparietal and limbic network showed a P+ > 0.90. Abbreviations: DMN = default mode network, FPN = frontoparietal network, SMN = somatomotor network, DAN = dorsal attention network, VAN = ventral attention network, LIM = limbic network, VIS = visual network. SUBC = subcortical structures.

On the mesoscale, there was moderate evidence for a negative association between percentage improvement and functional, but not structural connectivity of the DMN with the FPN, DAN and limbic network (P+ = 0.08–0.09; Supplementary Fig. 4). The association with DMN-FPN connectivity was most robust. Compared with non-responders, responders showed moderately credible evidence for lower functional connectivity between the DMN and FPN (P+ = 0.06) and DAN (P+ = 0.06) and lower structural DMN-FPN, DAN-VAN, DAN-FPN and VAN-SMN (P+ = 0.05–0.09) connectivity. There was strong to very strong credibility for associations of percentage improvement and response status with lower PC across all functional systems and particularly for the DMN, FPN, limbic and visual networks (all P+ <0.1; Fig. 2). There was only credible evidence for higher PC of the VAN (P+ = 0.94) and subcortical areas (P+ = 1) in responders. Multilayer eccentricity showed very strongly credible evidence for a negative association with percentage improvement (all P+ <0.05) but not response status across all systems (Fig. 3), while there was moderately credible evidence for higher multilayer eigenvector centrality in responders in the FPN (P+ = 0.91), DAN (P+ = 0.93), VAN (P+ = 0.92) and limbic network (P + −0.91). These results were generally robust to LOSO validation (Fig. 2, Fig. 3 and Supplementary Fig. 5–6). Exploratory NBS did not produce an interconnected subcluster associated with treatment efficacy.

### 3.4 Pre-to-post treatment changes

On the global level there were no significant associations between the change in single layer or multilayer network measures and treatment response, except that responders and non-responder differed in the pre-to-post-treatment change in small-worldness of the structural connectome (B[SE] = −61.2[25.9], P = 0.02) and multilayer eigenvector centrality (B[SE] = −1.2[0.6], P = 0.047; see Table 3). Non-responders showed an increase in small-worldness and eigenvector centrality, while responders showed a decrease in small-worldness but no change in eigenvector centrality. Adjusting for trial or treatment did not affect these results and there were no changes in other global measures (Supplementary Table 6–7).

**Table 3:** Pre-to-post treatment change in global connectome measures and treatment response.

	Crude model			Adjusted model*			LOSO	
% change	B [SE]	95 % CI	P	B [SE]	95 % CI	P	Trial (harm.P)	Treatment (harm. P)
Functional connectome								
Eglob (x 10^−2^)	6.100 [9.100]	−11.9|24.1	0.500	−	−	−	0.432	0.766
Q_yeo_ (x 10^−2^)	−5.800 [9.100]	–23.8|12.2	0.526	−	−	−	0.757	0.887
PC_yeo_ (x 10^−2^)	4.300 [9.100]	−13.7|22.3	0.638	−	−	−	0.866	0.982
Small-worldness (x 10^−2^)	−2.900 [9.100]	−20.9|15.1	0.750	−	−	−	0.879	0.997

Structural connectome								
Eglob (x 10^−2^)	9.200 [8.800]	−8.1|26.6	0.295	−	−	−	0.364	0.285
Q_yeo_ (x 10^−2^)	12.100 [8.800]	−5.2|29.4	0.170	−	−	−	0.165	0.042
PC_yeo_ (x 10^−2^)	13.700 [8.700]	−3.6|31	0.120	−	−	−	0.088	0.009
Small-worldness (x 10^−2^)	6.900 [8.800]	−10.5|24.3	0.434	−	−	−	0.65	0.717

Multilayer connectome								
EC (x 10^−2^)	−15.300 [9.200]	–33.5|2.8	0.097	−	−	−	0.055	0.003
Ecc (x 10^−2^)	4.500 [9.300]	−13.9|22.8	0.630	−	−	−	0.74	0.976

Responders vs non-responders								
Functional connectome								
Eglob (x 10^−3^)	0.200 [0.200]	−0.3|0.7	0.370	0.200 [0.200]	−0.3|0.7	0.369	0.425	0.489
Q_yeo_ (x 10^−2^)	0.200 [0.700]	−1.1|1.5	0.765	0.200 [0.600]	−1.1|1.4	0.763	0.973	0.998
PC_yeo_ (x 10^−2^)	−0.300 [0.500]	−1.3|0.8	0.637	−0.300 [0.500]	−1.3|0.8	0.635	0.908	0.967
Small-worldness (x 10^−2^)	−11.000 [11.800]	−34|12	0.353	−11.000 [11.700]	–33.8|11.8	0.350	0.518	0.485

Structural connectome								
Eglob (x 10^−4^)	0.000 [0.100]	−0.1|0.2	0.631	0.000 [0.100]	−0.1|0.2	0.627	0.836	0.951
Q_yeo_ (x 10^−2^)	−0.100 [0.400]	−0.8|0.7	0.872	−0.100 [0.400]	−0.8|0.7	0.872	0.734	0.999
PC_yeo_ (x 10^−2^)	−0.100 [0.200]	−0.4|0.2	0.491	−0.100 [0.200]	−0.4|0.2	0.492	0.74	0.78
Small-worldness (x 10^−2^)	−61.200 [25.900]	−111.7|−10.7	0.019	−61.200 [25.900]	−111.5|−10.9	0.019	0.001	<0.001

Multilayer connectome								
EC (x 10^−2^)	−1.200 [0.600]	−2.4|0	0.054	−1.200 [0.600]	−2.3|0	**0.047**	0.008	<0.001
Ecc (x 10^−2^)	−0.300 [0.600]	−1.5|0.9	0.580	−0.300 [0.600]	−1.5|0.8	0.580	0.715	0.943

*Corrected for age and sex. Abbreviations: LOSO = leave-one-sample-out – either one of the trials or one of the treatments. Eglob = global efficiency, Q = modularity quality index, PC = participation coefficient, EC = eigenvector centrality, Ecc = eccentricity.

Results on the mesoscale are reported in the supplements (Supplementary Figs. 7–11). NBS showed no interconnected subcluster that changed with treatment-induced symptom improvement or differed between responders and non-responders.

### 3.5 Post-hoc analyses

To account for possible medication effects, we added medication status as an additional covariate to the analyses associating the pre-treatment global topological measures and treatment response. Results remained, except for the small-worldness of the functional connectome which no longer showed a significant association (see Supplementary Table 8).

We performed Bayesian analyses with LOSO validation on the pre-treatment global topological measures to corroborate our NHST findings (Supplementary Figs. 12–13). These analyses showed highly credible and robust evidence for the reported associations with treatment efficacy. In addition, it showed high credibility for associations between percentage improvement and functional global efficiency (P+ = 0.03), average PC (P+ = 0.03), modularity (P+ = 0.96) and multilayer eigenvector centrality (P+ = 0.90), and similar results for responder versus non-responders.

Lastly, we repeated the analyses in the two OCD trials with five different treatments. Results are reported in the supplements (Supplementary Table 9). While effect sizes were similar as for the full sample, only the negative association between multilayer eccentricity and change in YBOCS reached statistical significance (B[SE] = −6.8[3.4], P = 0.048), presumably due to the small sample size.

## 4 Discussion

The aim of this study was to establish common connectome markers that are associated with successful treatment outcome across two psychiatric disorders (OCD and PTSD) encompassing nine different treatments from four clinical trials. Our results showed that treatment response was associated with several global measures of segregation and integration, primarily of the functional connectome, and an integration measure (eccentricity) of the structure–function multilayer. Using complementary statistical approaches, i.e. NHST and Bayesian (multilevel) modeling, we showed strong credible evidence for these associations. The results on the global level were largely mirrored on the mesoscale, showing credible evidence across all functional systems with particular involvement of the topology and connectivity of the DMN. The results were overall robust against leave-one-trial and leave-one-treatment out validation and medication status.

**Fig. 4:**
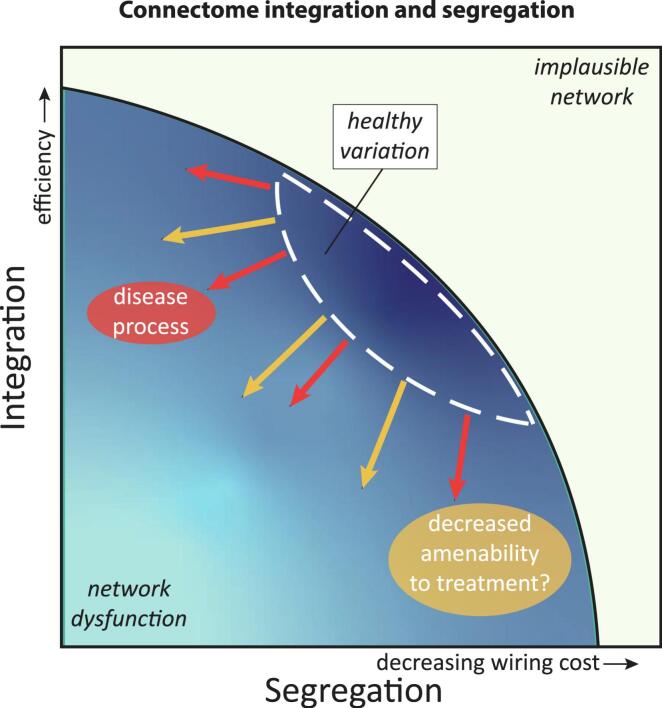
Schematic representation of the association between network topology and amenability to treatment. The architecture of the connectome is governed by the fundamental principles that under normal circumstances are in balance: segregation, i.e. tight local connections between brain areas that form a specialized subsystem, and integration, i.e. exchange between subsystems with long range connections. Deviation from the healthy variation in balance between these forces is associated with network dysfunction and the emergence of brain-related disorders as reviewed by (van den Heuvel and Sporns, 2019). The results of the current study suggest that (further) deviation from the norm is also associated with decreased amenability to non-pharmacological treatment for psychiatric disorders.

Individuals with the largest imbalance between integration and segregation (expressed as a lower small-worldness) of both the functional and structural connectomes benefited the least from treatment, as did individuals with lower functional modularity (i.e. lower modularity, higher average participation coefficient) and lower integrative capacity of the structure–function multilayer (eccentricity). Together these results suggest that individuals with brain connectomes that deviate more from a balanced and ‘healthy’ connectome are the least amenable to cognitive control enhancing treatments, regardless of the disorder and treatment (Fig. 4). This coincides with previous studies that identified modularity as a potential cross-disorder imaging marker for treatment response (Arnemann et al., 2015, Doucet et al., 2020, Gallen et al., 2016, Gallen and D’Esposito, 2019).

Individual differences in the brain’s capacity to *resist* pathological changes – regardless of what these are – has previously been conceptualized as a cognitive reserve (Stern, 2009), of which the neurobiological underpinnings are yet to be fully elucidated. Likewise, we postulate that the brain’s capacity to *benefit* from treatment can be conceptualized as a *Connectomic Reserve*: an intrinsic feature of someone’s brain network, regardless of the disorder, of which the interindividual variation determines amenability to treatment. Our current results suggest that a normal modular network structure and healthy balance between network segregation and integration is associated with greater benefits from treatment, but more research is needed to determine whether these topological measures can predict treatment outcome on the individual level.

We saw relatively few associations with treatment-induced changes in the connectivity and topology of the connectome, most of which were sensitive to LOSO validation. Both responders and non-responders showed an increase in multilayer eigenvector centrality after treatment, but this increase was higher in the non-responders. This was contrary to what we expected. Non-responders additionally showed larger decreases in structural connectivity and an increase in small-worldness after treatment compared with responders. An increase in average eigenvector centrality is indicative of higher integration and efficiency of the entire network. However, neither single layer global efficiency nor multilayer eccentricity, which are more direct measures of the efficiency of the network, showed such an effect. Instead, we speculate that these findings in combination with the decrease in system-to-system structural connectivity strength and increase in small-worldness may point towards a maladaptive shift in the hierarchical organization of the network. This maladaptive shift represents more local clustering between influential brain regions but at the cost of cross-modular long-range connections. The fact that on the system level we saw a relatively low robustness against LOSO validation, may stem from the smaller sample with pre-and-post-treatment MRI data but may alternatively suggest that the different treatments impact the connectome differently and increase variability.

Our findings further support previous reports showing common connectome dysfunction across psychiatric disorders (Lord et al., 2017, Sha et al., 2019, Spronk et al., 2021), by showing on average a less favorable functional and multilayer network topology and lower functional connectivity in psychiatric cases compared with healthy controls. Nevertheless, there was quite some variation, not only between individuals but also between trials and treatments and some findings were no longer significant when adjusting for trial or treatment (although the findings were robust against LOSO validation).

This study has a number of strengths. First, we used harmonized state-of-the-art MRI acquisitions with stringent quality control and a uniform processing pipeline. We additionally investigated different brain network modalities at multiple scales and used two complementary statistical approaches (NHST and Bayesian modeling), LOSO validation and two definitions for treatment response (percentage change, response status) to allow investigation of the robustness of the findings. Although there were some differences in the level of statistical significance or credibility of evidence between the percentage improvement and responder/non-responder models, with the exception of the multilayer eccentricity, they all pointed in the same direction. We currently do not have an explanation for the contrasting findings for multilayer eccentricity but this may stem from a difference in variance.

Some other limitations also need to be kept in mind when interpreting these findings. The current analyses were performed on the group level in two psychiatric disorders and it remains to be determined whether the findings extend to other psychiatric (and neurological) disorders, other (non-pharmacological) treatments and whether cross-disorder connectome measures can predict treatment outcome on the individual level. Let this manuscript therefore also be an invitation to all readers to collaborate and further test the existence of a universal Connectomic Reserve. The use of imaging data acquired on the same scanner with harmonized sequences and processing pipelines also prevented us from investigating robustness against differences in scanners and processing pipelines. Although adjusting for sex (and age) did not influence the results, there was a relatively higher predominance of females in this sample which may have influenced the results. The same is true for the higher proportion of individuals with OCD. Finally, the absence of harmonized sociodemographic measures beyond age and sex across the four clinical trials limited our ability to examine their potential impact on the results.

In conclusion, we showed for the first time that treatment outcome is related to transdiagnostic pre-treatment network topology across two psychiatric disorders and nine different non-pharmacological treatments. Specifically, we showed that individual variation in intrinsic features of the human connectome related to functional network modularity, integration/segregation balance and integration across the structure–function multilayer, underlies amenability to treatment. These results support a more dimensional approach towards precision medicine for psychiatric disorders with potential for network measures as cross-disorder biological markers for treatment response.

## CRediT authorship contribution statement

**Chris Vriend:** Writing – original draft, Visualization, Validation, Software, Project administration, Methodology, Investigation, Formal analysis, Data curation, Conceptualization. **Sophie M.D.D. Fitzsimmons:** Writing – review & editing, Data curation. **Inga Aarts:** Writing – review & editing, Data curation. **Aniek Broekhuizen:** Writing – review & editing, Investigation. **Ysbrand D. van der Werf:** Writing – review & editing. **Linda Douw:** Writing – review & editing, Methodology. **Henny A.D. Visser:** Writing – review & editing, Resources. **Kathleen Thomaes:** Writing – review & editing, Resources. **Odile A. van den Heuvel:** Writing – review & editing, Resources.

## Declaration of competing interest

The authors declare that they have no known competing financial interests or personal relationships that could have appeared to influence the work reported in this paper.

## Data Availability

Code is available from the author’s github page. Raw data falls under EU GDPR law but can me made available upon reasonable request.
